# A Literature Review on the Relative Diagnostic Accuracy of Chest CT Scans versus RT-PCR Testing for COVID-19 Diagnosis

**DOI:** 10.3390/tomography10060071

**Published:** 2024-06-14

**Authors:** Hafez Al-Momani

**Affiliations:** Department of Microbiology, Pathology and Forensic Medicine, Faculty of Medicine, The Hashemite University, Zarqa 1133, Jordan; hafez@hu.edu.jo

**Keywords:** COVID-19, computed tomography, chest CT, real-time polymerase chain reaction, RT-PCR, coronavirus, sensitivity, specificity

## Abstract

Background: Reverse transcription polymerase chain reaction (RT-PCR) is the main technique used to identify COVID-19 from respiratory samples. It has been suggested in several articles that chest CTs could offer a possible alternate diagnostic tool for COVID-19; however, no professional medical body recommends using chest CTs as an early COVID-19 detection modality. This literature review examines the use of CT scans as a diagnostic tool for COVID-19. Method: A comprehensive search of research works published in peer-reviewed journals was carried out utilizing precisely stated criteria. The search was limited to English-language publications, and studies of COVID-19-positive patients diagnosed using both chest CT scans and RT-PCR tests were sought. For this review, four databases were consulted: these were the Cochrane and ScienceDirect catalogs, and the CINAHL and Medline databases made available by EBSCOhost. Findings: In total, 285 possibly pertinent studies were found during an initial search. After applying inclusion and exclusion criteria, six studies remained for analysis. According to the included studies, chest CT scans were shown to have a 44 to 98% sensitivity and 25 to 96% specificity in terms of COVID-19 diagnosis. However, methodological limitations were identified in all studies included in this review. Conclusion: RT-PCR is still the suggested first-line diagnostic technique for COVID-19; while chest CT is adequate for use in symptomatic patients, it is not a sufficiently robust diagnostic tool for the primary screening of COVID-19.

## 1. Introduction

### 1.1. Review Background

Due to sustained human-to-human transmission, the coronavirus known as COVID-19, which originated in Wuhan City, China, has generated several clusters of pneumonia cases over recent years, spreading to more than 200 nations since December 2019. This process was worrisome enough to be considered a global public health emergency [1]. According to Gorbalenya and Baker [2], the clinical spectrum of COVID-19 includes instances that are asymptomatic, mild–to–moderate respiratory infections, severe cases that rapidly develop into acute respiratory distress syndrome (ARDS), and multiorgan failure with fatal results. Thus, early detection is critical in both symptomatic and asymptomatic cases in order to manage patients effectively and to implement the infection control measures needed to reduce the risk of spreading the disease to other members of society. Fever, dry cough, exhaustion, and the slow onset of dyspnea are the most typical clinical signs [3], while reverse transcription–polymerase chain reaction (RT-PCR) examination of respiratory tract tissue is the gold standard clinical method of diagnosing COVID-19 [4]. However, insufficient cellular material or mistakes in detection and extraction methods during nasopharyngeal swab sampling can result in significant rates of false negative findings for this test [5].

COVID-19 also takes 2 to 14 days to incubate, which means that an initial negative RT-PCR result cannot fully rule out a COVID-19 diagnosis [6]. Problems with specimen capture or errors made in the laboratory might also result in false negative results on using RT-PCR [7]. These tests must therefore be repeated in those patients for whom the initial RT-PCR test is negative who still exhibit clinical symptoms indicative of COVID-19 [8].

Various scientists have thus sought to explore the use of different modalities for the early diagnosis of COVID-19 infection, and previous studies have highlighted the role of chest CT scans in alternative diagnostic and screening procedures [9]. The primary factors contributing to the diagnostic utility of a CT scan of the chest are rapid scan duration and excellent lung lesion detection capability and categorization resolution. Furthermore, CT scanning offers a precise assessment of disease development, as well as being both incredibly easy to use and readily repeatable [10]. Early characterization of lung lesions, the evaluation of disease severity, and the improvement of lung lesions after therapy are the key benefits of chest CT in terms of identifying lung lesions in COVID-19 patients [11]. However, despite these advantages, no professional medical body recommends using chest CT as an early COVID-19 detection modality.

The aim of this review was thus to explore the role of CT scans in diagnosing COVID-19 infections, as well as to evaluate the sensitivity and specificity of chest CT scans as part of an exploration of their potential use as first-line diagnostic tools for COVID patients, replacing RT-PCR.

### 1.2. COVID-19: CT Imaging Manifestations

Numerous investigations have already been conducted on the CT imaging manifestations seen in instances of COVID-19. Peripheral ground-glass opacities (GGOs) and consolidation in the lower and middle lung regions, which are typically bilaterally distributed and multilobar, are the most prevalent chest CT imaging features in COVID-19 pneumonia [10,12,13,14]. According to Hefeda [12], some COVID-19 patients also have lung nodules on their initial CT scans that can be seen to enlarge and multiply on subsequent scans. According to Bernheim and Mei [15], the most frequent CT features that appear following symptom onset are consolidation, linear opacities, bilateral and peripheral disease, crazy-paving patterns, and reserved halo signs—all of which imply more extensive total lung involvement. On follow-up CT scans, patients with COVID-19 may also display rare abnormalities such as pneumothorax, pleural effusion, lung cavitation, lymphadenopathy, and pericardial effusion as their illness progresses [16].

Pathological alterations in the lungs are probably connected to these chest CT patterns in COVID-19 pneumonia [12,17], while angiotensin-converting enzyme 2 (ACE2) is a molecule that may have a role in the onset and course of acute lung failure in SARS patients [18,19]. ACE2 serves as both an enzyme and a functional receptor on the surfaces of cells, facilitating the entry of SARS-CoV-2 into host cells. It is also, unfortunately, abundantly present in the lungs. ACE2 plays a crucial role in controlling the renin–angiotensin–aldosterone system (RAAS), and SARS-CoV-2 disrupts the equilibrium between ACE/ACE2 and activates the RAAS, ultimately resulting in the advancement of COVID-19 [20]. In the lung, the impact of the disruption of the ACE/ACE2 system leads to pulmonary epithelial cell destruction, widespread alveolar damage, and edema, which might provide an explanation for the pathological underpinnings of consolidation and GGO, as well as the rapid alterations seen in chest CT imaging of COVID-19 patients [19].

### 1.3. COVID-19 Pneumonia: Early versus Late Chest CT Findings

CT scans of COVID-19 patients reveal that in various stages of disease progression, different features emerge in a discrete pattern [21,22]. Early on in the disease, patients present with a predominance of GGOs that vary in degree from mild to moderate. As the disease progresses, these GGOs consolidate to a degree and thus adopt a haphazard ‘crazy paving’ appearance. This is accompanied by deformation of the lung structures, subpleural and parenchymal bands, and limited vascular dilatation [21,22,23].

In their study of patients admitted to hospital with COVID-19 pneumonia, Zhou and Ren [24] used chest CT to track changes occurring in the lungs from the time of admission to follow-up. Based upon their observations, they determined that there was a specific pattern to the lung features that developed as the disease progressed. The primary feature observed on illness days zero to five was GGOs, and by days 6 to 11, GGOs were still present as a reticulated pattern emerged, though this was accompanied by lung consolidation. Between days 12 and 23, consolidation was less pronounced, while reticulation and the presence of simple GGO increased. These manifestations gradually resolved after day 24, before eventually disappearing. Lung abnormalities were at their severest on illness days 6 to 11, thus producing the highest CT score in this period.

These findings echo those reported by Kwee and Kwee [25], who determined that the patterns of COVID-19 observed in chest CTs followed four stages: In stage one, (days 0 to 5 after the onset of symptoms), the lungs appeared normal or presented with GGOs. The second stage, which covered days 5 to 8, was associated with progression, with the number of GGOs increasing and adopting a ‘crazy-paving’ appearance. The peak stage occurred 9 to 13 days after the onset of symptoms, typified by an increase in consolidation. The fourth stage began 14+ days after symptoms began. In this late stage, there was a resolution of consolidation and GGOs; signs of fibrosis might also emerge at this stage. The researchers also reported that the degree of lung abnormality and the temporal evolution of the disease did vary between patients, according to the severity of infection.

### 1.4. Differentiation of COVID-19 Pneumonia

It can be very difficult to distinguish between COVID-19 pneumonia and other viral or atypical pulmonary infections. Duzgun and Durhan [26] and Li et al. [27] stated that viral pneumonia most often involves interstitial lung tissue, and studies of common manifestations associated with the influenza virus include GGOs; small, nebulous nodules; and bilateral irregular consolidation [27,28]. However, the H1N1 and H5N1 strains of influenza present with rapid-progression pneumonia, which frequently results in acute respiratory distress syndrome (ARDS), which is not typically observed with common flu [29]. Ajlan and Quiney [30] described the features observed on CT in patients with H1N1 and H5N1 as including diffuse areas of GGO, cavitations, multifocal consolidations, lymphadenopathy, and pleural effusion. Whilst adults are typically unaffected by respiratory syncytial virus (RSV), this can be a problem for children and elderly people. RSV presents in an airway-centric pattern, with potential thickening of the bronchial wall and areas of ‘tree-in-bud’ opacity, with or without consolidation [27,31]. Pneumonia caused by cytomegalovirus (CMV) and herpes simplex/zoster virus (HSV/VZV) is also common among patients who are immunocompromised, and CT radiographs of these infections often show bilateral infiltration of the alveoli and interstitial tissue, with asymmetric areas of GGOs and areas of parenchymal consolidation. In patients with HSV, pleural effusion may be observed [32,33].

## 2. Methodology

### 2.1. Reviw Question, Keywords, and Databases

The review question was “What are the comparative diagnostic accuracies of chest CT and RT-PCR in terms of identifying and diagnosing COVID-19 infection in adults?” The following key words were used to search databases for the pertinent literature: “coronavirus”, “severe acute respiratory syndrome”, “SARS virus”, “COVID-19”, “SARS-CoV-2” AND “CT” OR “computerized tomography” OR “chest radiology” OR “thoracic radiology” OR “thoracic CT” AND “diagnosis” OR “sensitivity” OR “specificity”.

To locate papers relevant to the review question, the medical subject heading (MeSH) filter was used. To refine the search further, the Boolean operators “AND” and “OR” were used to retrieve papers where more than one search term applied, or where multiple keywords were used in one paper.

Having defined the review question and keywords, electronic databases, reference lists, and key journals were combed for studies addressing the review question. The Cumulative Index to Nursing and Allied Health Literature (CINAHL) and Medical Literature Analysis and Retrieval System Online (Medline) databases, provided by the Elton Stephens Company (EBSCOhost), were used. These databases cover the majority of scientific publications, though to ensure the search was comprehensive, the Cochrane (formerly referred to as the Cochrane Collaboration) and ScienceDirect catalogues were also interrogated. Finally, manual searches of reference lists and bibliographies were undertaken to ensure all relevant papers were identified. The reason for selecting these databases was that they were considered the most suitable repositories of papers relevant to the review question, and these databases did return a large number of papers, providing sufficient material with adequate data for this review.

Harris et al. (2014) highlighted the importance of establishing inclusion and exclusion criteria to direct a search strategy, as well as the need to ensure that the search is conducted to the highest possible standard to limit bias [34]. Only English-language articles published in peer-reviewed journals were thus included, and further inclusion criteria were applied, including the fact that the papers selected reported on studies of adults infected with COVID-19 who had simultaneously undergone chest CT and provided an RT-PCR test sample. The articles were also required to provide sensitivity and/or specificity statistics for these diagnostic methods. To broaden the population, the inclusion criteria did not distinguish between studies of exclusively symptomatic patients and those that considered both symptomatic and asymptomatic populations. The sex of patients was not considered a relevant factor.

All papers published in a language other than English were excluded. Other exclusion criteria included studies featuring children and those that did not specifically compare the findings of CT scans and RT-PCR. The review also excluded any study that recruited patients diagnosed as positive for COVID-19 solely based on RT-PCR results with no chest CT scan.

### 2.2. Database Search Result

Applying the keywords to filter the database search yielded 225 hits; 61 of these were duplicates that were removed. An inclusion/exclusion criterion screening review of the titles, abstracts, and conclusions was then performed on the remaining 164 studies. Of these, 118 studies were eliminated for failing to achieve the threshold for inclusion. Those excluded included (1) studies that examined the characteristic features of COVID-19 pneumonia as they appear on CT scans; (2) those where the topic of research was the development and evaluation of an automatic framework to be used with chest CT scans to detect COVID-19; (3) studies focused only on general CT findings in patients with COVID-19 pneumonia; (4) studies where, although the abstract was written in English, the body of the work was written in another language—typically Chinese or Italian; and (5) studies whose subject matter was not pertinent to the review question.

**Figure 1:**
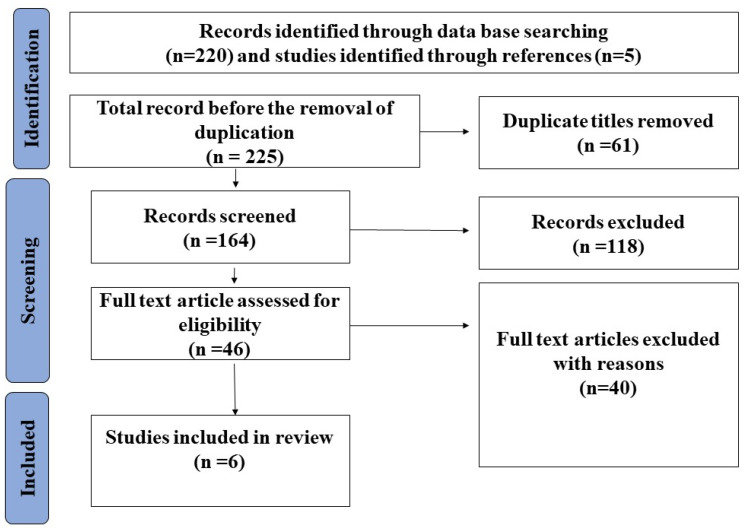
PRISMA chart for this review.

After applying the inclusion and exclusion criteria, sixty-six papers relevant to the review question remained. The full texts of these papers were extracted, though following close scrutiny of their content, sixty papers were eliminated for not comparing the diagnostic capabilities of CT scan and RT-PCR. Ultimately, six papers were identified as being relevant to the review. The PRISMA protocol was adopted throughout this process (Figure 1).

## 3. Findings

### 3.1. Assessment of the Included Studies

Each of the six papers included in this review presented its aims and objectives clearly. These were specific, and typically the aims included comparing the accuracy of chest CT and PCR with respect to diagnosing COVID-19. Three of the papers reported research conducted in China [35,36,37]. The remaining studies were conducted in Italy (*n* = 1) [38], Japan (*n* = 1) [39], and Turkey (*n* = 1) [40].

Each of the studies adopted a quantitative diagnostic accuracy study design and used a ‘single-gate’ testing approach, in which patients received an index test (CT scan) and a reference test (RT-PCR test). This approach is appropriate to the research question, as it creates a direct relationship between the two diagnostic modalities. Consequently, the results reliably reflect the diagnostic capabilities of chest CT with respect to COVID-19.

The cumulative total of patients providing data across the six studies was 2329. The largest cohort, over 1000 participants, was provided in Ai and Yang [35] (*n* = 1014), while the smallest cohort was 51 [36]. However, none of the studies justified the size of their samples. Due to the inherent relationship between the statistical precision of findings and sample size, quantitative analyses require large-sized samples. In addition to the study mentioned above [36], two other studies also had relatively small sample sizes (He and Luo [39], *n* = 82; Long and Xu [37]). These three studies’ small cohorts might thus be considered a limitation, casting doubt upon the reliability of their findings.

While Fang and Zhang [36] and Bellini and Panvini [38] adopted the consequent sampling approach, the other four studies used the convenience sampling method [35,37,39,40]. As indicated by its name, the convenience sampling method is a convenient non-probability method that relies on the availability of people prepared to take part in the study [41]. This method is popular because it is generally easy and cheap to recruit participants, but it has the drawback of an increased risk of sampling bias [42].

All studies included in this review, with exception of Ai and Yang [35], collected data from patients admitted to hospitals with moderate to severe symptoms. They did not include COVID-19 patients who were only mildly symptomatic or asymptomatic, which might have produced different results. Indeed, the diagnostic capabilities of CT might be exaggerated by the exclusion of mildly symptomatic and asymptomatic patients, as it is possible that making a diagnosis using CT in such patients would be rather more challenging. Consequently, the external validity of the studies may have been adversely affected, limiting the generalizability of the findings, and invoking participant selection bias.

An important risk of information bias evident in all the studies was the lack of blinding. In each patient, an RT-PCR test was administered, and the result was known prior to the conduction of the CT scan. The radiologists reading the CT scans were also not blind to the RT-PCR test result, and as they were not in an objective position, their analysis of the scans is likely to be vulnerable to bias. According to Kleinbaum and Sullivan [43], information bias, also known as misclassification, is a primary source of bias that undermines the validity of health research.

Only half of the reviewed studies stated the duration of the gap between conducting the RT-PCR test and performing the CT scan [35,36,38]. The durations noted ranged between two and seven days. The other three studies failed to mention the length of the intervening period between tests, creating a risk of disease progression bias occurring due to the duration between the tests being sufficiently extensive for the patient’s pathological state to change in that time. This could create a discrepancy between the disease state tested at baseline (RT-PCR) and the comparative modality (CT scan), which could manifest as the inflated accuracy of the latter. With time, particular characteristics of an acute disease can emerge or become more obvious, making it easier to identify those features.

Schmidt and Factor [44] highlighted another issue that arises from time lags between tests, which is that any therapy that the patient receives between the RT-PCR and CT scan may influence their disease state. Accordingly, a patient may improve (or deteriorate) due to therapeutic intervention, increasing the potential for bias.

### 3.2. Sensitivity and Specificity of the CT Scan Regarding the Detection of COVID-19

The six studies [35,36,37,38,40] evaluated the sensitivity and specificity of the CT scan modality in terms of its diagnostic accuracy for COVID-19. Table 1 displays the sensitivity of chest CT scans in terms of diagnosing COVID-19 across all of the examined studies. Sensitivity varied from 61% [38] to 98% [36]; the average was 69.4%. Specificity was provided in just four of the investigations, and this varied from 25% [35] to 96% [39], with an average of 68% seen throughout the studies.

Fang and Zhang [36] recruited 51 people (29 males and 22 females), and in each of these at least one RT-PCR test was conducted on a sputum sample (*n* = 6) or a throat swab (*n* = 45). Between the onset of symptoms and the results of the CT and RT-PCR testing, there was an average delay of three days (SD ± 3 days). Of the 51 patients, 36 returned a positive RT-PCR on the initial test, with 12 patients returning such a test within 1 to 2 days after the onset of symptoms: two patients were confirmed 2 to 5 days after symptoms emerged, while one patient only tested positive 7 days after becoming symptomatic. Of the 51 patients, 50 (98%; 95% CI: 90–100%) had features on their initial CT examination that suggested they had viral pneumonia. The number of positive CT diagnoses (50/51, or 98) was thus greater than the number of initial positive RT-PCR tests.

Long and Xu [37] assessed 36 individuals whose final diagnosis was pneumonia resulting from SARS-CoV-2 infection. At the time of hospital admission, 35 of these patients had abnormal CT scan results, while one subject had a normal scan. Thirty patients had a positive RT-PCR result, while the other six did not show any signs of infection. Of the latter group, three patients had positive results after three days, and the other three showed positive results after five to eight days. The sensitivities of diagnosis by CT and RT-PCR at the time of the initial evaluation were thus, respectively, 97.2% and 84.6%.

Ai and Yang [35] carried out research with a sizable cohort of COVID-19 patients (*n* = 1014); testing for viral nucleic acid on throat swab specimens was carried out alongside chest CTs and RT-PCRs. A positivity rate of 59% (95% CI: 56–62%) was obtained from the RT-PCR data, which yielded 601 positive and 413 negative results. Of the 601 RT-PCR positive patients, 580 had abnormal chest CT results. However, 308 (75%) of the patients with negative RT-PCR results had positive CT results. The RT-PCR assays and matching chest CT scans were separated by a median of one day (range: 0 to 7 days). Overall, 888 patients had abnormalities identified in chest CTs (88%; 95% CI: 86–90%) that demonstrated the diagnostic criteria for COVID-19 infection identification; as compared to the benchmark RT-PCR test, this gave results of a sensitivity of 97% (95% CI: 89–95%); a specificity of 25% (95% CI: 22–30%), and an accuracy of 68% (95% CI: 65–70%). Aslan and Bekci [40]’s study from 2021 assessed 250 people whose positive COVID-19 diagnosis was confirmed by RT-PCR. The initial chest CT scan had a sensitivity of 90.4% (95% CI: 86–93%) and a specificity of 64.2% (95% CI: 50–76%) as compared to RT-PCR. Ultimately, 226 individuals (90.4%) produced radiographic evidence of COVID-19.

Notwithstanding the encouraging results of earlier research [35,36,37,40], several methodological flaws exist that may compromise the validity of the conclusions—particularly in terms of overestimating the sensitivity of CT scans. Every participant in the Fang and Zhang [36] trial reported acute respiratory symptoms or pyrexia. The sensitivity of CT may thus have been exaggerated, as asymptomatic individuals were not examined in this investigation. Similarly, Long and Xu [37] calculated the sensitivity of chest CTs from a sample of 36 people, potentially leaving this artificially high due to the fact that they only included persons with either pyrexia (>38 °C) or potential pneumonic alterations. This study also had other acknowledged shortcomings: Due to a restricted supply of nucleic acid detection kits after a COVID-19 infection wave in the area, only patients with pyrexia and a positive CT scan were subjected to RT-PCR testing. In addition, the study’s population was both not-randomly assigned and small. Restricting samples to individuals exhibiting symptoms may thus limit the assessment of diagnostic criteria for CT generalizability to a larger population, as many COVID-19 cases are asymptomatic.

While Ai and Yang [35] reported a high sensitivity (97%) for CT identification of COVID-19, the actual sensitivity is likely to be lower than that of the RT-PCR reference test as their patient sample included only people who presented with pneumonia, creating a biased sample. Patients in critical condition were also more likely to be scanned early, which could affect the results of the study. Spectrum bias may also have resulted from the application of non-random sampling strategies, such as convenience sampling, as the research only included those thought likely to have the pathology.

On the other hand, research by He and Luo [39] and Bellini and Panvini [38] suggested that the CT scan method could only diagnose early-stage COVID-19 with a modest degree of sensitivity. He and Luo [39] included 82 individuals with potential COVID-19 who received baseline RT-PCR and chest CT testing at the same time. The reference test, the serial RT-PCR, yielded positive results in 34 patients and negative results in 48. Sensitivities for the diagnosis of COVID-19 were thus 79% (95% CI: 66–93%, *n* = 27) and 77% (95% CI: 100%, *n* = 48) for the first RT-PCR and first chest CT scan, respectively. With accuracies of 92% (95% CI: 91–92%) and 88% (95% CI: 88%), respectively, the specificities for RT-PCR and chest CT were 100% (95% CI: 66–93%, *n* = 27) and 96% (95% CI: 90–100%, *n* = 46), respectively.

Bellini and Panvini [38] determined the extent of lung disease brought on by COVID-19 using the COVID-19 Reporting and Data System (CO-RADS) CT severity score. Twelve radiologists with different levels of experience scored each CT separately, and three groups of scan readers were created based on high, middle, and low expertise levels. The three reader populations had corresponding sensitivity values of 69.1% (95% CI: 62–77%), 63% (95% CI: 54.2–69.9%), and 62.7% (95% CI: 53.2–70.1%). Readers with the greatest and lowest experience levels produced specificities of 84.1% (95% CI: 81–87%) and 79.3% (95% CI: 75.1–83%), respectively. As a result, not only was chest CT sensitivity deemed poor in terms of identifying COVID-19, but it was also seen to vary depending on the expertise and experience of the CT scan reader. There were no asymptomatic participants in the trial; however, as all symptomatic patients who reported to an emergency care with probable COVID-19 were included in the study cohort, it is consequently possible that the study’s patient recruiting strategy was skewed in favor of participants who were more seriously ill, which might have affected the results of the CT scan accuracy estimates.

## 4. Discussion

The purpose of this review was to evaluate the diagnostic accuracy of chest CT scans as compared to RT-PCR tests in terms of diagnosing COVID-19. A total of six studies that investigated the sensitivity of chest CTs regarding the diagnosis of individuals with COVID-19 were evaluated and analyzed.

The sensitivity of chest CT scans with respect to COVID-19 diagnosis was recorded in all examined studies. This varied from 61% [38] to 98% [36]. The average sensitivity across studies was 69.4%. Compared to RT-PCR assays, chest CT scans were generally assessed as having a higher sensitivity, according to four studies [35,36,37,40]. The other two studies, however, found that CT had a lower sensitivity than RT-PCR, making it insufficient for the initial detection of COVID-19 in patients.

There are fewer statistics available concerning the specificity of chest CT in terms of detecting COVID-19 than there are on the sensitivity of CT more generally. Specificity was reported in just four of the studies examined, for an average of 68% across all trials; this varied from 25% to 96%. Ai and Yang [35] and Aslan and Bekci [40] reported that the specificities of chest CT scans in identifying COVID-19 were 25% and 64.2%, respectively. This produced further inconsistency with the other two studies, which reported a moderate-to-high specificity for CT scans, ranging from 87.3% [38] to 96% [39].

There are several plausible explanations for the discrepancies in the statistical measures employed across publications. These include differences in factors such as the CT scan procedures employed, sample sizes, the inclusion criteria utilized for recruitment, and the expertise levels of the scan readers. Bellini and Panvini [38] hypothesized that discrepancies may be related to the scan reader’s seniority and thus their expertise in analyzing CT scans. This is important in many facilities, as an initial scan is commonly read by radiologists who are still in training. Nonetheless, such staff should still be able to recognize common COVID-19 symptoms from radiographs.

The findings of this literature review are in agreement with the systematic review and meta-analysis undertaken by Khatami and Saatchi [45]—their work evaluated the accuracy of chest CTs for diagnosing COVID-19. The reported sensitivity and specificity were 87% (95% CI: 85–90%) and 43% (95% CI: 29–63%), respectively. In another meta-analysis, Kovács and Palásti [46] explored chest CT sensitivity across sixteen studies, calculating it to be 92%. The researchers noted, however, that only two of the sixteen studies provided specificity data, which emerged at 25% to 33%. A third meta-analysis reported the combined sensitivity and specificity of chest CTs to be 94% and 37%, respectively [47].

The quantitative studies included in this review were diagnostic accuracy studies with third level hierarchical evidence. Nonetheless, these studies had some methodological limitations: Some of the studies were at risk of patient selection bias, as they did not include asymptomatic or mildly symptomatic patients, nor did they adopt a randomized or consecutive sampling process. Three of the studies [36,37,40] did not discuss what steps, if any, were taken to blind the readers to the results of the RT-PCR tests; therefore, these studies must be considered at risk of bias with respect to the results of the CT scans. Furthermore, all of the studies included selected patients presenting with specific clinical features, such that the disease symptoms were of significant severity that the patients underwent chest CT to assess their clinical situation. As patients in the early stage of COVID-19 infection might not have developed clinical features detectable by CT scan, such as GGOs and respiratory consolidation, this offers the potential for the modality’s sensitivity to be exaggerated.

Another source of bias is that these studies were carried out during the COVID-19 pandemic. The prevalence of the disease at the time increased the pre-test probability of COVID-19, reducing the prospect of alternative diagnoses. Were the same studies to be repeated now or in the future, it is possible that other diseases, such as influenza, RSV, and various other respiratory infections, would be seen as prima facie being responsible for such illness. In such instances, the specificity of CT chest scans would be reduced further, making it an ineffective diagnostic modality.

Despite the CT features of COVID-19 being widely reported, it can still be difficult to differentiate COVID-19 from other forms of viral pneumonia [48]. The features present on CT scans of patients with COVID-19 infections appear similar those present in other viral pneumonias [49]. GGOs are a common feature with the earliest presentation; however, such features are not unique to COVID-19. As described by Koo and Lim [50], GGOs are common features in many viral pneumonias. Furthermore, the same radiographic patterns that occur bilaterally in the inferior lobe in COVID-19 are also common in other viral pneumonias [51,52].

To complicate matters further, the presence of GGOs is not exclusive to viral infections, being instead associated with diverse lung conditions, air-space diseases, alveolar collapse, and interstitial thickening [32]. GGOs can reflect the thickening of the alveolar walls or interstitium, or be caused by cells of fluid accumulating in the alveolar spaces. Mild cases of GGOs are especially difficult to identify radiographically, and, traditionally, GGOs were considered a non-specific feature. All these points could help explain the low specificity of CT scans in the diagnosis of COVID.

Furthermore, to establish whether chest CT is a suitable COVID-19 screening modality, several factors must be taken into account. To consider it as a first-line screening tool for a large population, it is particularly important to determine the risk–benefit ratio of using chest CT. Ribeiro and Husson [53] state that at ~0.6 mSv/year, medical imaging is the largest man-made source of radiation exposure. The standard dose of chest CT is around 1.8 mSv. According to Kang et al. [54], low-dose chest CT (~0.2 mSv) is effective for identifying COVID-19 infection. Nonetheless, if chest CT were used routinely in large populations to diagnose COVID-19, the population overall would be exposed to a significant increase in radiation. In addition, chest CTs could pose an increased risk of disease transmission. Although the provision of dedicated protection devices should not increase the risk of transmission any more than conducting a swab test, as CT equipment is not disposable, it must be cleaned thoroughly after each use. In fact, to eliminate the risk of nosocomial transmission of COVID-19, all equipment must be cleaned carefully and there should be an adequate air flow in the room [55].

Furthermore, the measures needed to eliminate nosocomial transmission take time. It takes 35 to 45 min to remove 99% of airborne contaminants effectively in CT suites with 6 to 8 air changes per hour. Cleaning the equipment also takes around 35 to 45 min, meaning that a single CT suite can only process one or two patients an hour [55,56].

Based on the evidence from reviewing the literature, it is clear that, as a primary screening method, chest CT scans are neither suitable nor effective. The recommended primary diagnostic tool thus continues to be RT-PCR. However, to limit the number of false negative RT-PCR results, respiratory tract samples must be collected from suspected COVID-19 patients with care.

The review’s findings align with the American College of Radiology’s position that chest CTs should not be used as a screening method or as a first line of diagnostic inquiry in patients who may have COVID-19 [57]. The Centers for Disease Control (CDC) agency also maintains that RT-PCR is the only valid diagnostic approach for SARS-CoV-2 infection, and the organization this does not currently recommend plain X-ray or chest CT [58]. Even though the radiological evidence for COVID-19 is extremely suggestive, verification is thus required in each case. This is due to the fact that the features shown in CT and X-ray scans are not specific to COVID-19; they can also be brought on by influenza, H1N1, SARS, and MERS infections, among other viral diseases. The specificity of CT is further limited by the fact that influenza is more likely to be encountered at its peak time than in cases of COVID-19 [59].

In a statement released on March 12, 2020, the Royal College of Radiologists (RCR) in the United Kingdom advised against using CT as part of the diagnostic process for people with potential coronavirus infections. After two weeks, however, the RCR changed its mind and recommended that chest CT be used to estimate progression and complication among critically sick patients. The Royal Australian and New Zealand College of Radiology (2021) further endorsed the approach of not using CT as a screening tool, while the Canadian Association of Radiologists affirmed in March 2021 that a normal chest CT scan could not rule out a diagnosis of COVID-19—particularly in cases where the patient has had a short symptom duration [60]. Similarly, as it is the gold standard test for coronavirus presence, the WHO still advises the use of RT-PCR testing.

Despite the limitations of CT scans in terms of the early diagnosis of COVID-19 infections, Polak and Van Gool [61] claim that CT could be a useful tool for developing understanding of the pathology associated with disease progression. Furthermore, it could play a role in identifying concurrent or alternative diagnoses, as well as in detecting possible complications of COVID-19 pneumonia. According to Rabiee and Eibschutz [62], the preferred imaging tool for assessing the state of a patient’s lungs following COVID-19 infection is high-resolution CT (HRCT), while follow-up studies conducted by Vijayakumar and Tonkin [63] and Bocchino and Rea [64] have attempted to characterize the pulmonary changes observed on CT scans. These pulmonary parenchymal abnormalities persist for some time; among the manifestations observed on CT scans up to a year following infection are GGOs, ‘crazy paving’, parenchymal bands, reticulations, septal thickening, and traction bronchiectasis/bronchiolectasis. Infrequent honeycombing has also been detected. Yasin and Gomas [65] and Stewart and Jacob [66] suggest that chest CT might be a useful tool to use in the recovery period to monitor the lungs for recovery as well as various COVID-19 complications, sequelae, and the development of lung disease. Armed with knowledge concerning the range of appearances of the lung following COVID-19 infection, radiologists may thus be able to make more accurate diagnoses, which in turn will help in delivering timely and appropriate treatments to future patients.

## 5. Conclusions

Based upon the literature reviewed in this study, both RT-PCR and CT scans have limitations in terms of diagnostic accuracy for COVID-19. Chest CTs have limited specificity in terms of identifying COVID-19 pneumonia, while the procedure for detecting nucleic acids using RT-PCR is susceptible to contamination. Four studies included in this review demonstrated that RT-PCR assays are highly sensitive in detecting COVID-19, but they also suggest that chest CT scans may have even higher sensitivity. However, it is important to note that all of these studies had methodological flaws, which means that the available data on the high sensitivities of diagnostic tests for COVID-19 may be imprecise. Furthermore, two further investigations contended that CT scans should be employed only in individuals displaying symptoms. In the context of screening or early diagnosis, the findings of this review thus do not support the use of CT scans, based on prior recruitment of symptomatic patients only. The review also identified a number of limitations associated with using CT scans being used to diagnose COVID-19 infection, offering minimal evidence to support using this modality in a diagnostic capacity.

This review itself has a number of limitations, however. The first is that the review only considered six studies, none of which achieved high-quality evidence. The scope of the review was constrained by only including English language studies; thus, any non-English language reports of studies containing valuable data were lost. Furthermore, this criterion might have introduced some reporting bias. The inclusion criteria and the number of databases searched might also have limited the review, and while identifying suitable search terms was done through a carefully considered process, the selected search terms might have filtered out other works that may have been relevant. Finally, the sample size of some of the studies was very small, which might have skewed their findings, failing to reflect the situation across a wider population.

## Figures and Tables

**Table 1:** Summary of the findings presented in each study (N/R means ‘not reported’).

	Sensitivity of Chest CT (95% CI)	Sensitivity of the First RT-PCR Test	Specificity of CT Scan	Specificity of RT-PCR
Fang and Zhang [36]	98% (90–100%)	71% (56–83%)	N/R	100%
Long and Xu [37]	97.2% (N/R)	84.6%	N/R	100%
Ai and Yang [35]	97% (95–98%)	N/R	25% (22–30%)	100%
Aslan and Bekci [40]	90.4% (86–93.7%)	51.6%	64.2% (50.3–76.6%)	100%
He and Luo [39]	77% (62–91%)	79% (66–93%)	96% (90–100%)	100%
Bellini and Panvini [38]	61% (52–69%)	N/R	81% (77–84%)	100%

## Data Availability

The datasets used and/or analyzed during the study are available from the corresponding author on reasonable request.
